# In vivo quantitative imaging of photoassimilate transport dynamics and allocation in large plants using a commercial positron emission tomography (PET) scanner

**DOI:** 10.1186/s12870-015-0658-3

**Published:** 2015-11-09

**Authors:** Abhijit A. Karve, David Alexoff, Dohyun Kim, Michael J. Schueller, Richard A. Ferrieri, Benjamin A. Babst

**Affiliations:** Biological, Environmental, and Climate Sciences Department, Brookhaven National Laboratory, Upton, NY 11973 USA; Present address: Purdue Research Foundation, West Lafayette, IN 47906 USA; Present address: Five Eleven Pharma Inc, Philadelphia, PA 19104 USA; Present address: School of Forestry and Natural Resources, University of Arkansas at Monticello, Monticello, AR 71656 USA

**Keywords:** Carbon allocation, Positron emission tomography (PET), Transport, Imaging, Carbon-11 (^11^C)

## Abstract

**Background:**

Although important aspects of whole-plant carbon allocation in crop plants (e.g., to grain) occur late in development when the plants are large, techniques to study carbon transport and allocation processes have not been adapted for large plants. Positron emission tomography (PET), developed for dynamic imaging in medicine, has been applied in plant studies to measure the transport and allocation patterns of carbohydrates, nutrients, and phytohormones labeled with positron-emitting radioisotopes. However, the cost of PET and its limitation to smaller plants has restricted its use in plant biology. Here we describe the adaptation and optimization of a commercial clinical PET scanner to measure transport dynamics and allocation patterns of ^11^C-photoassimilates in large crops.

**Results:**

Based on measurements of a phantom, we optimized instrument settings, including use of 3-D mode and attenuation correction to maximize the accuracy of measurements. To demonstrate the utility of PET, we measured ^11^C-photoassimilate transport and allocation in *Sorghum bicolor*, an important staple crop, at vegetative and reproductive stages (40 and 70 days after planting; DAP). The ^11^C-photoassimilate transport speed did not change over the two developmental stages. However, within a stem, transport speeds were reduced across nodes, likely due to higher ^11^C-photoassimilate unloading in the nodes. Photosynthesis in leaves and the amount of ^11^C that was exported to the rest of the plant decreased as plants matured. In young plants, exported ^11^C was allocated mostly (88 %) to the roots and stem, but in flowering plants (70 DAP) the majority of the exported ^11^C (64 %) was allocated to the apex.

**Conclusions:**

Our results show that commercial PET scanners can be used reliably to measure whole-plant C-allocation in large plants nondestructively including, importantly, allocation to roots in soil. This capability revealed extreme changes in carbon allocation in sorghum plants, as they advanced to maturity. Further, our results suggest that nodes may be important control points for photoassimilate distribution in crops of the family Poaceae. Quantifying real-time carbon allocation and photoassimilate transport dynamics, as demonstrated here, will be important for functional genomic studies to unravel the mechanisms controlling phloem transport in large crop plants, which will provide crucial insights for improving yields.

**Electronic supplementary material:**

The online version of this article (doi:10.1186/s12870-015-0658-3) contains supplementary material, which is available to authorized users.

## Background

It has been estimated that, to meet world food demand crop production must double by 2050 [1, 2]. Plant growth and yields are dependent on photosynthetic fixation of carbon, and optimal allocation of carbon to growing sink tissues. Thus, in order to achieve this increase in yields on available arable land, we must develop a mechanistic understanding of photosynthetic C-fixation, C-transport and C-allocation to different tissues, and the regulatory system that controls photosynthesis and C-allocation [3]. It has been estimated that as much as 50 to 80 % of the C assimilated from CO_2_ through photosynthesis in a mature “source” leaf is exported out of the leaf to satisfy the demand of the non-photosynthetic “sink” tissues of the plant [4]. Experimental manipulations suggest that C-fixation and sink utilization are tightly co-regulated [5, 6]. For example, a decrease in sink demand reduces sugar transport from the leaves which results in sugar accumulation in the source leaves, enhanced expression of genes involved in carbohydrate metabolism, and decreased expression of photosynthetic genes [7, 8]. In contrast, increased sink demand enhances photosynthesis [6]. In soybean, root nodulation by N-fixing *Bradyrhyzobium japonicum* which increases C-demand of the roots results in elevated leaf photosynthesis [5]. It has been shown that growing plants under elevated atmospheric CO_2_ initially results in higher photosynthetic rates, but eventually is followed by a down-regulation of photosynthetic activity presumably due to the negative feedback resulting from inherently limited sink capacity [9–11]. Based on these observations it has been hypothesized that maintenance of high photosynthetic rate is dependent on the rate of carbon utilization and/or capacity for carbon storage of sink tissues. The cross-talk between the source and sink is facilitated in part by the regulation of phloem transport from source to sink, however we still do not fully understand the mechanisms underlying phloem transport. In part, this has been due to limited availability of technologies to measure phloem transport dynamics (e.g., photoassimilate transport velocity). Improving our understanding of the mechanisms that drive phloem transport may lead to new approaches for manipulating photoassimilate allocation.

Imaging tools that measure photoassimilate transport and allocation in plants non-destructively on appropriate spatial and temporal scales will facilitate investigations of the genetic, biochemical, and physiological mechanisms underlying C-transport and C-allocation [12]. Positron emission tomography (PET) is a functional imaging technique that can be used to determine whole-plant transport and nutrient allocation by quantifying the distribution of a positron emitting radioisotope in a plant non-invasively, over a time-course, and with a spatial resolution of a few millimeters. In addition to the radioisotopes carbon-11 (^11^C) and nitrogen-13 (^13^N), most of the plant nutrients have positron-emitting isotopes that could be detected using PET. By measuring allocation and transport of essential elements, such as carbon and nitrogen, in a whole plant in real time, PET provides a key tool needed to identify and characterize the mechanisms and regulation of transport and allocation. Also, the high sensitivity of PET is ideally suited for studies of rapid changes in plant function in response to treatments (e.g., hormone or environmental treatments, or developmental changes). Traditional carbon-14 (^14^C) and carbon-13 (^13^C) techniques are destructive, and the root compartment and thick tissues are essentially inaccessible for imaging. Like X-ray-computed tomography (CT) and magnetic resonance imaging (MRI), PET can image roots in soil and within thick stems, but PET can also provide spatially explicit and dynamic imaging of photoassimilate and nutrient transport in the root and stem. Several PET scanners have been developed specifically for plants recently [13–17], including a combined PET/CT scanner [18], and combined PET/MRI [19]. Although these PET imaging systems are designed for use with plants, most plant biologists do not have access to an instrumentation team to develop and operate them, and these experimental systems are usually scaled for small plants.

Studies of large plants are uniquely important because much of our food is produced as seed late in development, during the reproductive stage. For example, in the top three staple crops in the world (maize, rice, and wheat), the seed or grain is the only part of the plant edible to humans. Previous studies suggest that the mechanisms controlling photoassimilate uptake into the edible portions of the plant may be fundamentally different than in vegetative meristems at the shoot or root tips [20]. Therefore, studies of photoassimilate transport and allocation in large crop plants at the reproductive stage are important to devise new strategies for improving crop yields.

Here, we describe the use of a commercially available clinical PET scanner for functional imaging of ^11^C-labeled photoassimilate in plants. This presents a feasible option to make the technology accessible to many more plant biologists, since many universities have commercial PET scanners for medical research programs. Although the application of PET imaging to plants is relatively recent, PET has been used extensively for human and animal research and diagnostic imaging for over 30 years. The commercial clinical PET scanners have high spatial resolution (~3 mm) and high sensitivity, and have streamlined and user-friendly image processing packages, including automated spatially explicit measurement of radiation attenuation by the plant tissues and soil [21]. Further, the clinical PET scanners have a large bore, a large field of view (15 cm long × 55 cm diameter FOV), and computer controlled bed that enable measurement of dynamics in large plants up to ~ 1.5 m in height. We demonstrate this PET imaging approach using *Sorghum bicolor* (sorghum). Sorghum is a member of the family Poaceae (the grasses), which also includes the top three world staple crops, maize, rice and wheat. Sorghum is one of the top ten food crops in the world, and one of the most important staple crops for people in impoverished regions of Africa and Asia.

## Results and discussion

**Fig. 1:**
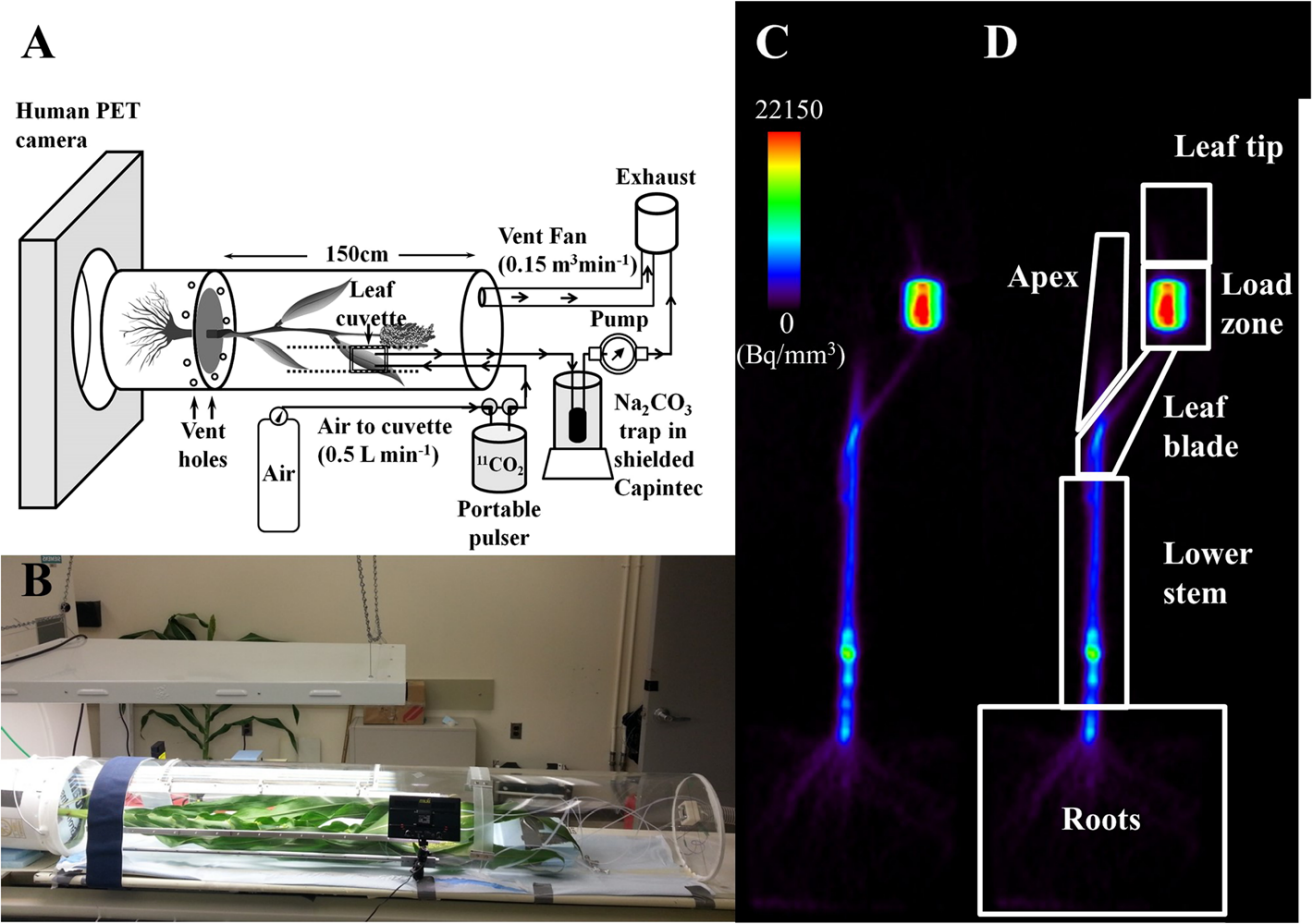
Experimental set up of sorghum for PET imaging using a commercial clinical PET scanner. **a** Schematic of the ^11^CO_2_ administration and imaging system developed for large grasses. **b** Side view of a 70 day-old- plant used in one of the experiments, the black rectangular box in the picture is the LED light panel used to ensure consistent illumination of the leaf cuvette. **c** A reconstructed PET image of ^11^C distribution in 70 day-old- sorghum-plant. **d** PET image shown in **c** with ROIs drawn to measure ^11^C-allocation to different tissues

In order to simultaneously administer ^11^CO_2_ to the plants and study ^11^C transport and allocation with a commercial PET scanner we developed a portable handheld ^11^CO_2_ pulsing system to deliver ^11^CO_2_ from the cyclotron to the PET scanner [22], and an externally illuminated plexiglass chamber that enclosed the whole plant to maintain environmental control, to keep the plant in position as it moved through the PET scanner, and to provide secondary containment of ^11^CO_2_, much like a fume hood (Fig. 1). We acquired dynamic images of ^11^C distribution in sorghum stems immediately after the ^11^CO_2_ pulse using a commercial PET scanner, and a static image of ^11^C-allocation 2 h later (Fig. 1c, d).

### Quantitative considerations for imaging plants with a clinical PET scanner

Commercial clinical PET scanners are calibrated using large uniform cylindrical ^18^F or ^68^Ge/^68^Ga phantoms of known radioactivity for imaging human subjects [23]. In addition to calibration, quantitative PET measurements require several corrections to the raw data that compensate for either misidentified scattered coincidences or “missing” data due to photon attenuation [24]. Robust methods to correct for these well-understood phenomena have been validated and are included with all clinical PET cameras [24]. The extent of these data corrections is dependent on the size, shape and composition of the object being imaged. For this reason it is a standard practice to calibrate clinical PET cameras with phantoms whose geometry and composition is representative of the human body (like a large cylinder filled with water). In contrast, the geometry of plant tissue presents an extreme range of morphologies whose dimensions vary greatly from the human body. In large grasses like sorghum the aerial part of the plant consists of a cylindrical stem whose diameter is about 3 cm, leaving most of the imaging FOV filled with air. Roots on the other hand are relatively smaller in diameter and are buried in soil. Because of this extreme difference in image object dimensions and composition from those normally used with a clinical PET camera, one goal of this study was to estimate the attenuation and scatter factors for different plant tissues for an accurate measure of radioactivity. The HR+ PET scanner used here has an automated procedure using built-in radioactive sources to determine attenuation and scatter (see methods). To test and optimize PET scans of plants, we attached ^18^F-phantom vials inside the stem of a sorghum plant (70 DAP), and inside the soil/root compartment at different depths (Additional file 1). We determined attenuation and scatter of the phantom without radioactivity, and measured the ^18^F radioactivity of the phantom vials with the PET scanner to compare with measurements of the same phantoms in a calibrated γ-scintillation counter.

**Fig. 2:**
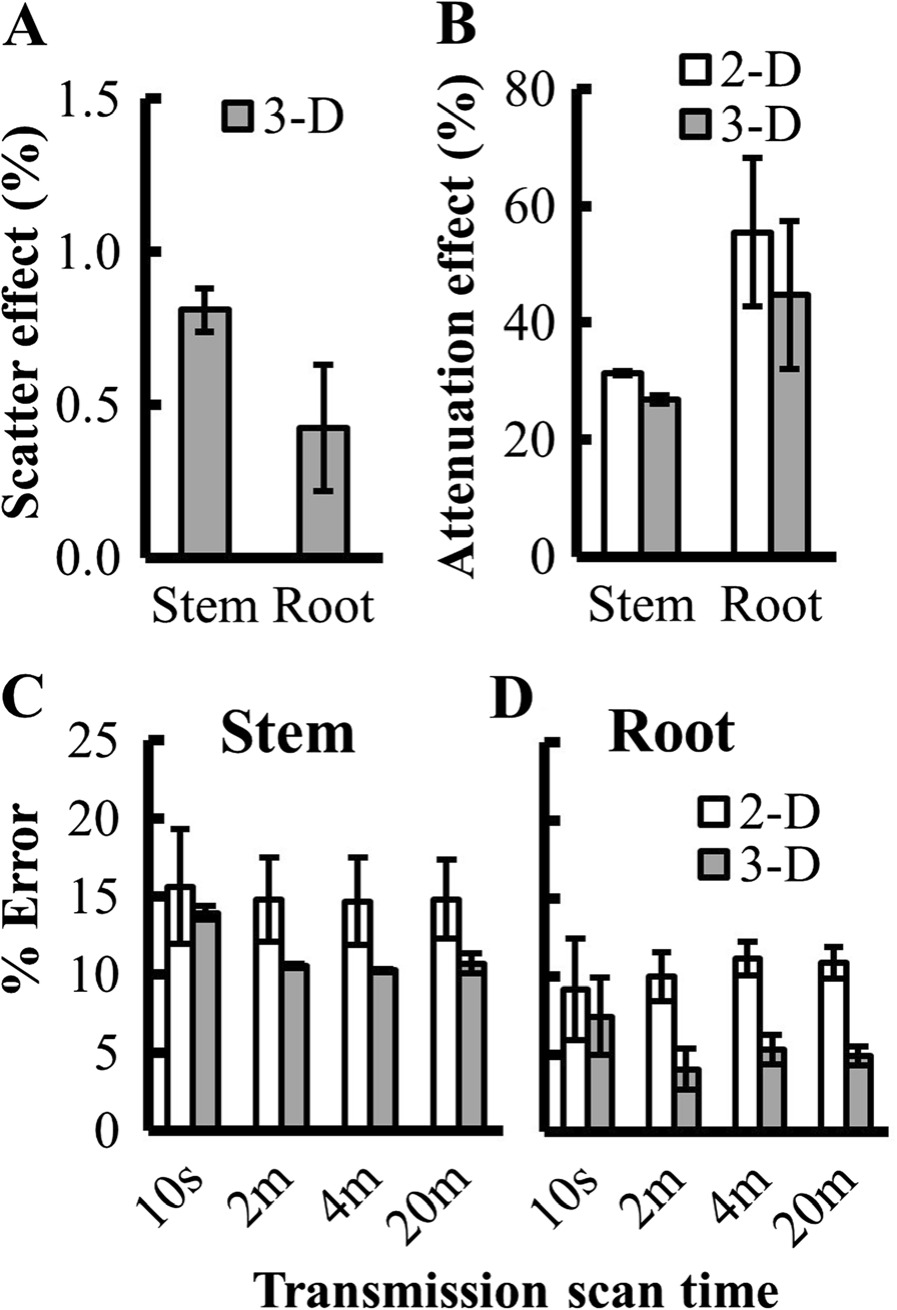
Optimization of PET parameters using ^18^F plant phantoms. **a** Effect of scatter on the accuracy of stem and root phantom radioactivity measurements. Bars show percent reduction in the error of phantom measurement after scatter correction under 3-D mode. **b** Effect of attenuation on the accuracy of stem and root phantom radioactivity measurements. Bars show percent reduction in the error of phantom measurement after attenuation correction under either 2-D (white) or 3-D (Grey) mode**. c** & **d** Error in the stem **c** and root **d** phantom radioactivity measurements based on the emission scans under 2-D and 3-D mode with different transmission scan times (10 s, 2 min, 4 min, and 20 min). For (C) and (D), bars show the average error of PET measurements of ^18^F phantom radioactivity, after attenuation correction and scatter correction, as a percent of the radioactivity of the phantoms measured using a calibrated scintillation counter. For all graphs error bars represent standard error

First we examined the effect of scatter on the accuracy of radioactivity measurements. The number of scattered coincidence events depends on the thickness and density of the object being imaged. Because the sensitivity to scattered coincidences is expected to be greater in 3-D mode than in 2-D mode, we measured the effect of scatter only in 3-D mode. Scatter correction had little effect (<1 %) on the shoot and root phantom radioactivity measurements (Fig. 2a).

To evaluate plant and soil attenuation of radioactivity, we performed transmission scans, in which each of the detectors in the PET scanner ring measured the radioactivity transmitted through the sample (i.e., the plant) from known radioactive source rods (^68^Ge). From transmission scans, the ECAT software generates a spatially resolved attenuation map that can then be used to correct for attenuation by the different tissues of the plant. We determined the phantom radioactivity with and without attenuation correction. There was a substantial reduction in the coincidence counts when we did not correct for attenuation (data not shown). Attenuation was the source for about 30 % error in stem and 55 % error in root radioactivity measurements (Fig. 2b). Since it is clearly important to use attenuation correction to quantify radioactivity in plants accurately, we optimized the transmission scan time by performing transmission scans on the plant phantom for 10 s, 2 min, 4 min and 20 min after the ^18^F radioactivity decayed below detection limits. The accuracy in the measurement of root and stem phantoms was affected by the transmission scan time. The average error was highest for the 10 s transmission scan (Fig. 2c, d). Increasing the transmission scan time to 2 min reduced the error to 10 % for the stem and 4 % for the root phantoms in 3-D mode (see below for explanation of 2-D and 3-D modes). However, increasing transmission scan time beyond 2 min did not result in a further reduction in error (Fig. 2c, d). Our analysis suggests that attenuation correction with the transmission scan of 2 min can significantly improve the accuracy of PET measurements. Therefore, we used attenuation correction in all further studies with at least 2 min transmission scans for stem segments, and 4 min transmission scans for the root segments, since there was slightly higher attenuation by the dense soil surrounding the roots.

We compared the accuracy of measurements in both 2-D and 3-D modes. Positron emitters, such as ^11^C, decay via emission of a positron which, upon collision with an electron, emits two 511 keV γ-rays in opposite directions [25]. Taking advantage of these coincident gamma rays, PET scanners incorporate multiple rings of individual gamma ray detectors to provide positional information (line of response; LOR) when two coincident photons are detected within a narrow time window. Most commercial scanners can be operated in either 2-D mode, which counts coincidences only within the most closely situated rings of detectors, or 3-D mode, which allows coincidence counts between any of the detector rings. For our phantom study, the emission scans were performed in both 2-D and 3-D mode. Overall, 3-D mode was more accurate for estimating radioactivity of the plant phantoms and was slightly more sensitive compared to 2-D (Fig. 2c, d). Because of higher accuracy and inherently higher sensitivity of 3-D emission scan, we used 3-D mode for acquiring emission data in our sorghum study.

### Measuring transport dynamics in sorghum

**Fig. 3:**
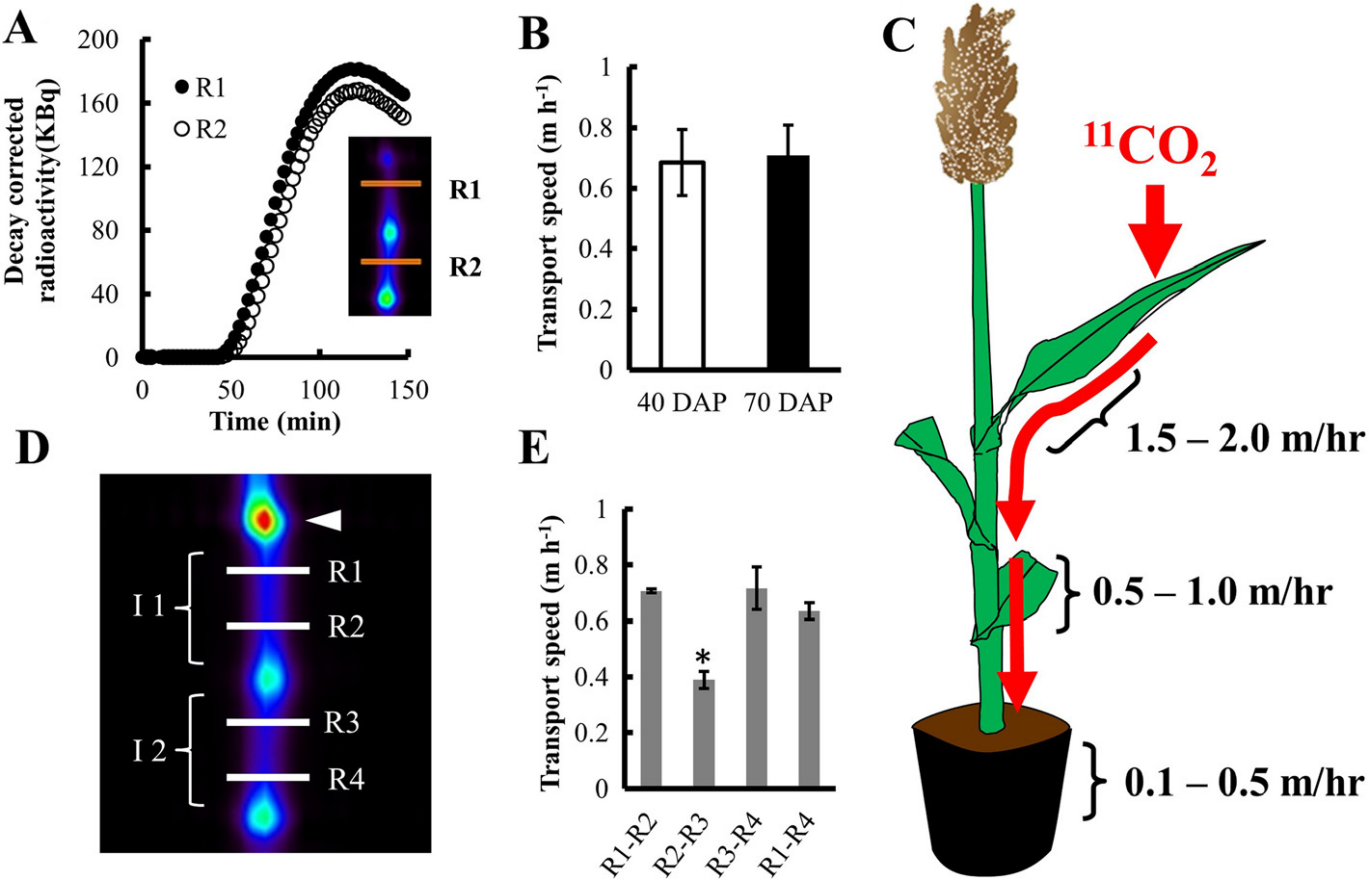
Use of PET to measure the dynamics in photoassimilate transport. **a** Changes in decay corrected radioactivity over time of ^11^C at two ROIs (R1 and R2) shown in the insert. Each curve represents changes in radioactivity over time in that ROI. The high radioactivity (i.e., bright green) spots in the insert are nodes. **b** Transport speed of the ^11^C-photoassimilate between R1 and R2 measured at 40 and 70 DAP in sorghum, bars are mean ± SE of at least 3 replicate plants. **c** ^11^C-photoassimilate transport speeds measured in leaf blades, stems, and individual roots. **d** Reconstructed image of the scanned sorghum stem in the FOV of the PET scanner with the individual ROIs (R1, R2, R3 and R4) outlined to estimate the changes in transport within an internode or across a node. White arrowhead shows the point of attachment of the target leaf; I1 and I2 are the two internodes in the FOV. **e** Transport speeds between different ROIs, calculated from TOAs using a combination of two of the ROIs, shown in (**d**)

Using a commercial PET scanner, we measured transport of photoassimilate in plants at two developmental stages: 40 days after planting (DAP) and 70 DAP. The 40 DAP plants had 5–7 fully developed leaves and were 70–90 cm tall; the 70 DAP plants had 10–12 fully developed leaves and were 100–120 cm tall. Each plant was positioned with lights on and left untouched for 1 h prior to ^11^CO_2_ administration to the target leaf. We acquired dynamic emission data for 2 h (in 2 min bins) from the internode just below where the target leaf sheath connected to the stem (for movie see Additional file 2). On the reconstructed, decay-corrected image, we outlined two ROIs of the same size (40 mm × 40 mm × 4 mm) so that each ROI encompassed the girth of the internode. The first ROI (R1, Fig. 3a, see inset) was near the top of the internode close to the point of attachment of the target leaf and the second ROI (R2) was outlined on the next adjoining internode. From the time-activity plot for each ROI (Fig. 3a) the time of arrival (TOA) of the ^11^C-photoassimilate was estimated (see methods) [26], to calculate transport speed as distance between the two ROIs divided by the time of ^11^C transport between the two ROIs. The average transport speed of the newly fixed photoassimilate was 0.7 m h^−1^ in stems of both 40 DAP and 70 DAP sorghum plants (Fig. 3b). Although laying a plant horizontal will certainly affect plant function over the long-term, there was little or no effect of the horizontal positioning required for using the commercial PET scanner on transport speeds, ^11^C fixation (75-80 %), or photosynthetic CO_2_ exchange rates (measured with an IRGA) compared with vertical plants within the 3 h time frame of this experiment (Additional file 3). To avoid potentially confounding gravitrophic effects, PET scanning of plants should be completed within 7–8 h of laying the plant horizontally, when carbohydrate utilization begins to change [27]. Our transport speed measurements are similar to previously reported transport speeds in stems at the base of small sorghum plants, although plants in that study were grown hydroponically [28]. Interestingly, we found that transport speeds were not constant throughout the plant. The ^11^C-photoassimilate transport speed was highest within the leaf blade (1.5 – 2 m h^−1^), and decreased substantially in the stem and further still in the roots (Fig. 3c). This decrease in speed along the axis of transport may be a consequence of photoassimilate unloading along the phloem pathway in the stem and roots.

**Fig. 4:**
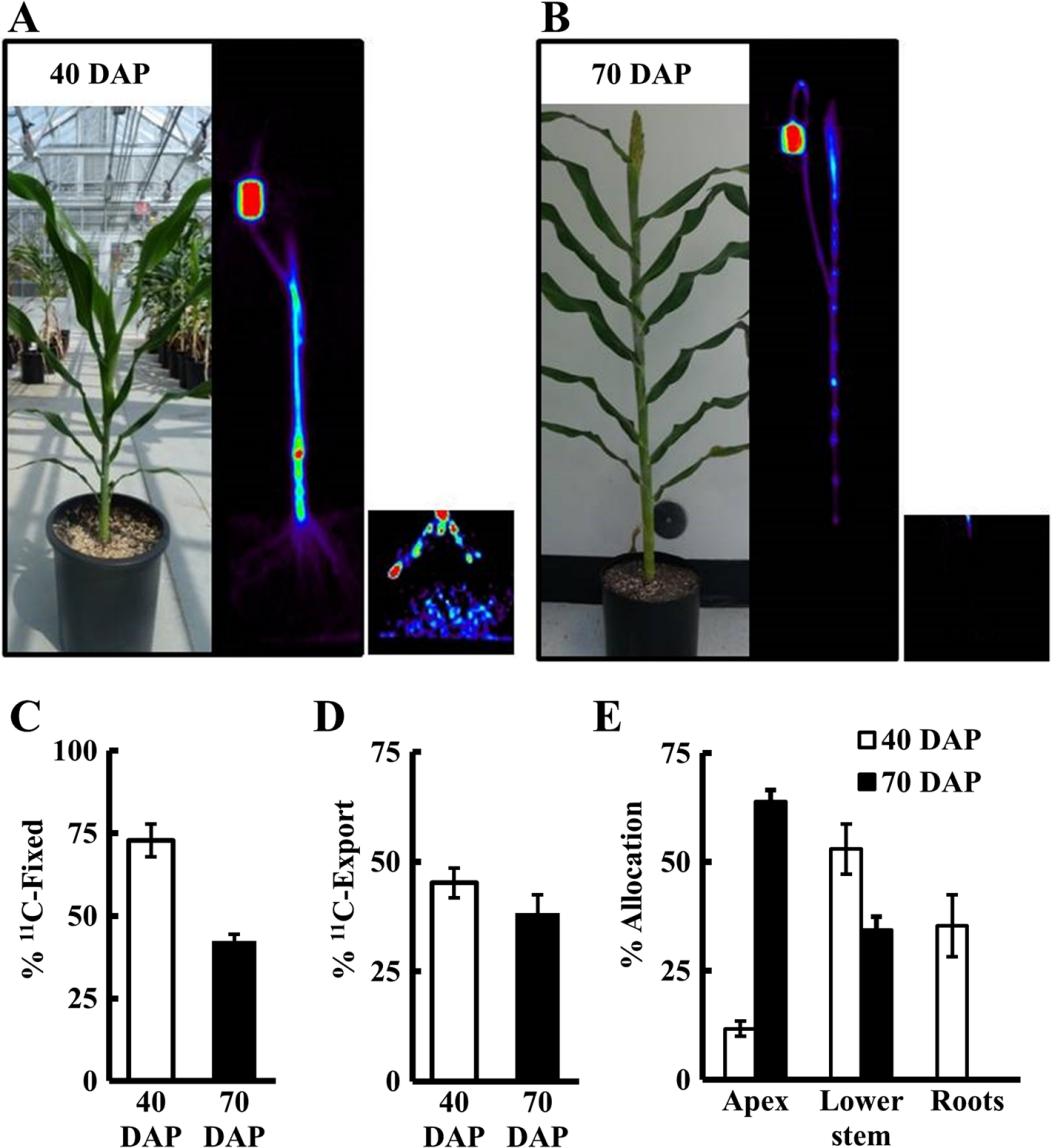
Use of PET to analyze C-allocation in sorghum at two developmental stages. Two hours after the administration of a 1 min pulse of ^11^CO_2_ to the youngest fully expanded leaf of a sorghum plant, excluding the flag leaf, the entire plant was scanned in 15 cm segments. Representative PET images of sorghum plants at, **a** vegetative stage (40 DAP) and **b** early flowering stage (70 DAP), showing distribution of ^11^C. Insets show roots with higher contrast for visualization, with contrast set the same level in A and B. **c** Percent fixation determined based on the total ^11^C administered minus the ^11^C radioactivity not fixed, measured in a soda lime trap placed inside a Capintec Dose Calibrator at 2 min after pulsing. **d** Percent export from the target leaf, and **e** percent allocation of exported ^11^C to roots, lower stem, and apex. The data represent the mean ± SE of 3 independent plants

Previously, we observed accumulation of ^11^C-labeled photoassimilate in nodes of maize, a close relative of sorghum (data not shown), suggesting that nodes may be a region of heavy phloem unloading. Since substantial phloem unloading in the nodes could affect photoassimilate flow across the nodes, we compared transport speeds between different sets of ROIs both within an internode and across nodes. We outlined four ROIs at ~25 mm intervals, two in the target internode (R1 and R2), and two in the internode below it (R3 and R4) within the FOV (Fig. 3d). ROIs could be placed closer together than ~25 mm, in theory as close 3 mm, but as the space between ROIs gets smaller, the time bins of data acquisition (2 min per data point here) need to be reduced to provide appropriate time resolution. Within an internode, the average transport speed was about 0.70 m h^−1^ between R1 and R2, and between R3 and R4. The transport speed across the node depended on the distance between the ROIs (Fig. 3d). The transport measured across R1 to R4 was 0.63 m h^−1^, which is slightly lower compared to transport speed measured within the internodes. Interestingly, the transport speed between R2 and R3, the two closest ROIs across the node, was significantly lower than the others (Fig. 3e). The nodes of monocots particularly may act as strong sinks due to the presence of intercalary meristem [29]. The nodes in large sorghum plants might act similarly to the reported “discrimination center” at the base of smaller sorghum plants [28], since high radioactivity is apparent at the nodes in our sorghum images (Figs. 1c, 3d, 4a, b), and similarly at the nodes of maize (data not shown). Substantial phloem unloading at the nodes could possibly explain the slowing of the photoassimilate transport speed across the nodes, but would not explain why faster speeds are resumed basipetal to the node. Alternatively, the complex anatomy at the nodes may contribute to the actual slowing, or appearance of slowing, in transport speeds. First, there may be substantial redirection of photoassimilate from basipetally-flowing to acropetally-flowing pipelines [30], which, like unloading, could possibly slow the transport speed. Second, flow velocity through a tube decreases proportionally with increasing tube cross-sectional area. Total sieve-tube cross sectional area is larger within the nodes than internodes of monocots [31]. This larger cross sectional area would result in slower transport speed or velocity, since flow velocity through a pipe is inversely proportional to pipe cross-sectional area. This would also explain why the transport speed increases again basipetal to the node, where sieve tube cross-sectional area becomes smaller again. Finally, the vascular bundles do not follow a straight path through the nodal plexus [32]. Our measurement of distance was the straightest path, which should be somewhat less than the actual length of the meandering path followed by the vascular bundles through the node. This underestimated distance would yield a calculated transport speed that is slower than the actual transport speed through the vascular bundles in the node.

### Measuring whole-plant carbon allocation

We also used PET to measure changes in C-allocation as the plants transitioned from a vegetative (40 DAP) to a reproductive (70 DAP) stage (Fig. 4a, b). This developmental shift in sorghum and in most grasses is characterized by higher C-allocation to the apex as the flower head begins to develop, and by initiation of internode elongation [33]. We targeted ^11^CO_2_ administration to the upper most (i.e., youngest) fully expanded leaf excluding the flag leaf in both the 40 DAP and 70 DAP plants. To measure allocation, we scanned the entire plant in 15 cm segments, applied attenuation correction (transmission scan performed for each plant prior to ^11^CO_2_ administration), and used the reconstructed whole-plant image to measure ^11^C- distribution to the target leaf, lower stem, apex and roots (ROIs as indicated in Fig. 1d). The target leaf was split into three ROIs, leaf tip, load zone and the leaf blade (Fig. 1d). The load zone, the region of the target leaf where the leaf cuvette was attached to administer ^11^CO_2_, generally had the highest radioactivity (50-65 %). Overall, the young sorghum plants (40 DAP) fixed more ^11^CO_2_ than the older plants (70 DAP; Fig. 4c). This was consistent with lower overall photosynthetic CO_2_ fixation by 70 DAP as measured by IRGA in a greenhouse (data not shown). A similar decrease in leaf photosynthesis with age has been previously reported by two separate studies in sorghum [34, 35]. The export from the target leaf was estimated by subtracting the radioactivity in the target leaf from the total radioactivity in the entire plant. The percent of fixed ^11^C exported from the target leaf did not change significantly between 40 DAP and 70 DAP (Fig. 4d), although the total C exported decreased from 40 to 70 DAP due to the decline in C-fixation. However, the allocation to specific tissues changed significantly as the plants matured. In particular, the younger plants allocated more ^11^C to the roots (Fig. 4e), while the older plants allocated a higher proportion of ^11^C to the apex (~60 %) and almost no ^11^C to the roots. The allocation to the lower stem was higher in the younger plants (~55 % of the total ^11^C-exported) than in the older plants (35 %; Fig. 4e), consistent with a transition from stem growth at the vegetative stage to flower head development at the reproductive stage. Source-sink dynamics within the plant determines how much, when, and where the carbohydrates are allocated which ultimately affects yield and productivity. Here, sink demand in stems declined during flowering, which may be a trait that has been selected to the extreme in domesticated crops, such as grain sorghum, as an unintended consequence of breeding for high grain yields.

**Fig. 5:**
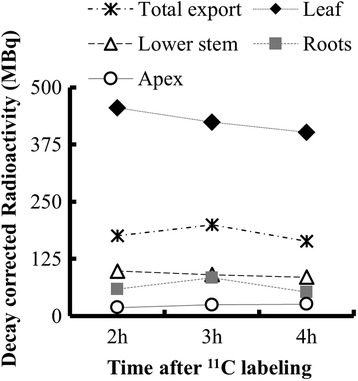
^11^C allocation at different times after ^11^CO_2_ administration. Quantification of ^11^C-allocation from three emission scans performed 2 h, 3 h, and 4 h after ^11^CO_2_ administration to the leaf of a 70 DAP sorghum plant. Each data point represents decay-corrected ^11^C-radioactivity (MBq) measured in that ROI, except for total export which represents total radioactivity exported from the leaf for each scan

To test how long we can measure C-allocation with the routine administration of ~50 mCi (1.85 GBq), three successive emission scans were performed on the same 70 DAP sorghum plant, 2, 3 and 4 h after administering ^11^CO_2_. The measure of export from the target leaf was higher at 3 h than at 2 h, with a corresponding reduction in the estimate of ^11^C radioactivity in the target leaf (Fig. 5). After 4 h, radioactivity in the target leaf decreased further, suggesting that more ^11^C was exported since 3 h. However, the amount of radioactivity detected in the rest of the plant after 4 h was lower than at 3 h, which was largely due to decreased radioactivity detected in the roots. Overall, the root zone had the lowest concentrations (ranging from 0.2-15 kBq mm^−3^) of radioactivity at both growth stages in sorghum. In order to visualize the roots, we had to increase the contrast above that needed for optimal visualization of the stem. The highest radioactivity was in the young adventitious roots near the top of the soil and the mesh of young fine roots near the bottom of the pot (Fig. 4a inset). Most likely, after 4 h the concentrations of radioactivity in some regions of the root were near or just below the detection limits of the PET scanner. However, the commercial PET scanner provided good estimates of ^11^C-allocation up to 3 h after radioactivity was administered. This was more than enough time to assess whole-plant C-allocation patterns, which were apparent by 2 h and changed only slightly between 2 and 3 h.

## Conclusions

Large plant size is an important challenge to studying C-allocation in large grasses such as *Sorghum bicolor*, particularly later in plant development when understanding of C-allocation is crucial for improving grain yields or composition of bioenergy feedstocks. Our results show that, by optimizing certain parameters and accounting for geometry of plants, commercial PET scanners can be used reliably not only to measure C-allocation in plants but also to study dynamics in photoassimilate transport. One advantage of PET is that it provides both quantitative and spatial information on C transport and allocation in intact undisturbed roots in a 3-dimensional soil column, which is not possible by other means, showing regions of high carbon demand, which generally correlates with high metabolic activity. Hence, PET provides functional imaging that can complement the spatially resolved imaging provided by other modalities, such as CT and MRI. In addition to studying developmental changes, this technology could also be used to compare species, genotypes, or a variety of environmental treatments to understand the genetic mechanisms regulating C transport and allocation. Furthermore, because the technology is non-destructive and the ^11^C and ^13^N radioactivity decays below the level of detection within 6–12 h, the same individual plant can be repeatedly pulsed and imaged over many days to measure the individual response to a treatment or developmental transition. This PET imaging method is suitable for imaging large grasses such as corn and sorghum; however, it also could be used for imaging smaller plants or other large plants (e.g., poplar). This method could also provide a platform for studying transport of phytohormones and nutrients that can be labeled with positron-emitting isotopes (e.g., ^11^C, ^13^N, ^18^F), which may also play important roles in reproductive development in large maturing plants.

## Methods

### Plant material and growth conditions

Seeds of grain sorghum (cultivar Macia; provided by Dr. Ismail Dweikat, University of Nebraska, Lincoln) were planted in 15 L pots containing a potting mix composed of Promix-BX ®: sand: perlite mixed in 2:1:1 (v/v). The potting mix was supplemented with 8 g of Osmocote ® (The Scott’s Miracle Gro Company, Marysville, OH) per pot at the time of planting. Plants were grown in the greenhouse maintained at 30 °C/24 °C day/night temperature cycle, under a 16 h photoperiod. Thirty days after planting (DAP), 3 g of Miracle-gro all purpose plant food (24-8-16) (The Scott’s Miracle Gro Company) was applied to each pot once per week until the plants reached maturity. Prior to the administration of ^11^CO_2,_ plants were transferred to a growth chamber (Conviron Inc. Winnipeg, Canada) maintained at 27 °C/24 °C day/night temperature cycle under a 16 h photoperiod, and 650 μmol m^−2^ s^−1^ photosynthetically active radiation (PAR) for 3d to acclimate to lab conditions.

### Generation of ^11^CO_2_ and administration to the sorghum plants

Carbon-11 was produced at BNL via the ^14^N(p,α)^11^C nuclear transformation using 17 MeV protons in a TR-19 Cyclotron (Ebco Industries Ltd, Richmond, BC, Canada) [36]. A 2760 kPa N_2_ gas target with 500 ppm O_2_ (99.9995 % purity gases; MG Industries) was irradiated with a 17 MeV proton beam for 1–2 min, depending on the amount of radioactivity desired. The resulting ^11^CO_2_ was captured on a molecular sieve and was either used directly for pulse administration to sorghum as previously described [37] or transferred to a portable pulser for the transport of ^11^CO_2_ to the PET facility as described previously [22]. For secondary containment, a plexiglass tube (0.3 m diameter × 1.8 m length) was customized with an adjustable leaf cuvette mounted inside the plexiglass tube on metal rails and a door panel to access and manipulate the plant (Fig. 1a, b). Before an experiment, the pot containing the sorghum plant was placed inside a 19 L bucket (Fig. 1a), and the surface of the soil was covered with 2.5 cm styrofoam insert to secure the soil in place. The shoot of the sorghum plant was gently placed inside the plexiglass tube lying horizontally on the bed of the PET scanner, and the rim of the bucket was fitted snuggly into the plexiglass tube. Although the chamber constrained the leaves and caused some self-shading, sorghum is generally planted densely enough in the field that leaves often overlap each other and cause self-shading, especially for lower leaves. The 19 L bucket had holes to allow air inflow, and the other end of the plexiglass tube (closed end) was connected to an exhaust fan (0.15 m^3^ min^−1^), conceptually much like a fume hood, in order to supply a continuous and adequate flow of air to maintain ambient air temperature and humidity around the entire shoot, and to provide secondary containment for ^11^CO_2_ gas supplied via the leaf cuvette. The plexiglass tube was illuminated externally by fluorescent light bulbs to provide 250 μmol m^−2^ s^−1^ photosynthetically active radiation (PAR) uniformly along the stem of the sorghum plant (Fig. 1b). The trap within the portable pulser was heated to 300 °C to release the ^11^CO_2_ from the molecular sieves, and then valves were switched to flow the leaf cuvette supply air through the trap for pulse administration. ^11^CO_2_ was administered as a 1 min pulse in a continuous air flow from a compressed air tank (ambient air with ~ 385 ppm CO_2_), with a flow rate of 0.5 L min^−1^ to a single leaf inside the plexiglass leaf cuvette (internal volume 0.09 L). The ^11^CO_2_ addition to the airstream made a negligible contribution to overall CO_2_ (<1 ppb). An array of adjustable LED lights (0–1000 μmol m^−2^ s^−1^ PAR) was positioned with fixed geometry relative to the leaf cuvette, with light intensity adjusted to 700 μmol m^−2^ s^−1^ PAR at the leaf surface. After performing a transmission scan, the plant was left undisturbed in position, with lighting on for 1 h prior to ^11^CO_2_ administration. The outlet of the leaf cuvette was connected to a soda lime trap positioned inside a Capintec radioisotope dose calibrator, to measure the amount of ^11^CO_2_ that was not fixed by the leaf. To ensure no outward leak of ^11^CO_2_ from the leaf cuvette gasket-leaf interface, an air pump was connected to the outlet tubing after the soda lime trap and adjusted to maintain the air pressure slightly below atmospheric pressure during ^11^CO_2_ administration. The air pump was switched off 2 min after ^11^CO_2_ administration and the airflow through the cuvette was maintained at 0.5 L min^−1^. The radioactivity in the Capintec was recorded at this time and was used to calculate ^11^CO_2_ fixation (^11^CO_2 administered_ – ^11^CO_2 not fixed_ = ^11^CO_2 fixed_).

### PET scanning

The PET scans were performed using an HR + PET scanner (Siemens, Germany) operated by ECAT (version 7.2.2) image acquisition and analysis software. For all ^11^C PET scans performed on the sorghum plants, we measured transport dynamics of ^11^C in a fixed location on the stem for 2 h after ^11^CO_2_ administration, and then scanned the entire plant to measure allocation of recently fixed carbon to the different plant tissue. Before isotope administration and emission scanning of ^11^C, a transmission scan was performed for each plant to correct for attenuation. During automated transmission scans, shielded radioactive source rods (^68^Ge) positioned around the PET scanner ring are sequentially exposed to measure transmission of radioactivity from that fixed point through the subject to each of the detectors on the opposite side of the scanner ring. This transmission scan procedure also provides a measure of gamma ray scatter caused by the subject. Routinely, plant set-up was completed at least 30 min prior to running the transmission scan. In order to acquire dynamic images, the region of interest - generally the subtending internode - was placed inside the 15 cm field of view (FOV) of the PET scanner by adjusting the bed position. Transport speeds were determined based on the dynamic imaging. On the reconstructed, decay-corrected dynamic images, we outlined two ROIs of the same size, encompassing the girth of the internode. From the time-activity plot for each ROI (Fig. 3a) the time of arrival (TOA) of the ^11^C-photoassimilate was estimated similar to the method described by Suwa and colleagues [26]. Briefly, the TOA was determined by identifying the slope at the inflection point of the time-activity curve, and extending the line back to determine at what time (TOA) it intersected with the baseline radioactivity. Transport speed was calculated as (distance between the two ROIs) / (TOA_1_ - TOA_2_). After scanning the subtending internode for 2 h, an automated program adjusted the bed position to scan the entire plant in 15 cm segments for 3 min each with an overlap of 7 cm between two adjacent segments, selected based on preliminary experiments. The length of the segment and overlap was always the same for both transmission and emission scans. The transmission scan for the whole plant allocation was performed for 2 min/segment on the shoots and 4 min/segment on the roots. The transmission scan for the dynamic imaging of the subtending internode was performed for 5 min. Images were reconstructed in ECAT and the reconstructed image files were analyzed using AMIDE (version 1.0.4) [38], which provides quantification of radioactivity for ROIs drawn by the user.

Time of the transmission scans was optimized with ^18^F-phantoms. A known quantity of ^18^F-fluoride was pipetted into 5 ml plastic vials and the volume was adjusted to 5 ml with water. For the stem phantoms, a small portion of stem was removed with a scalpel blade and the 5 ml vial containing ^18^F-fluoride was placed in this cavity (Additional file 1). The vials were secured to the stem with a plastic wire tie. For the root phantoms, the vials with ^18^F-fluoride were placed near the roots of the same plant inside the potting mix (Additional file 1). The plant with the ^18^F-phantoms was placed inside the plexiglass plant imaging tube and emission scan performed as described above. After 15 h for decay of ^18^F below background, transmission scans were performed on the plant phantoms. Images for the phantom studies were reconstructed with or without attenuation or scatter correction as described for each experiment. The error in the radioactivity measurement of plant phantoms was estimated by comparing radioactivity measurement in the phantom vial obtained from the PET scanner with that obtained from a calibrated γ- scintillation counter (Picker international, West Plains, NY, USA) equipped with Quantum MCA nuclear spectroscopy software (Princeton Gamma-Tech, Inc., Princeton, NJ, USA).

## Additional files

Additional file 1:
^**18**^ **F phantoms used for analysis of attenuation and scatter factors.** (A) The arrangement of ^18^F phantoms on the stem of the sorghum plant and inside the soil. (B) Image of the phantoms reconstructed from the PET emission scan. (C) ROIs outlined on the reconstructed image for quantification of radioactivity in each phantom vial. (JPG 377 kb) Additional file 2:
**Projection movie showing changes in** ^**11**^ **C-radioactivity over 120 min in two internodes of a sorghum plant inside a PET scanner.** (MP4 1480 kb) Additional file 3:
**Horizontal positioning has limited effects on physiological parameters of sorghum.** (A) A/Ci curve of sorghum plant kept upright (vertical) or after laying it down (horizontal) for 2 h. The photosynthetic CO_2_ assimilation [A] was measured using a LI-6400XT portable photosynthesis system (Li-Cor, Lincoln, NE). All the measurements were performed at 2000 μmol m^−2^ s^−1^ light intensity in a leaf cuvette maintained at 30 °C. Internal CO_2_ concentration [Ci] of the leaf cuvette was programmed to adjust from 0–700 μmol mol^−1^ of CO_2_, at a flow rate of ~500 ml min^−1^, and relative humidity between 50-60 %. Data was recorded on the first fully expanded leaf (youngest leaf with collar fully open). (B) Transport speeds of vertically and horizontally positioned 40 DAP sorghum plants. For sorghum plants in the horizontal position, transport speeds were determined as described in the methods. For sorghum plants in the upright position, ^11^CO_2_ was administered as described previously [37]. Briefly, two γ-radiation detectors with collimated lead shielding were positioned on the stem of the sorghum below the point of attachment of the load leaf and data was acquired for 2 h. The transport speed was determined from the time-activity curve generated for each detector as previously described, dividing the distance between the two detectors by the time of transit of [^11^C]-photoassimilate between the two detectors [26, 39]. (JPG 188 kb)

## Abbreviations

A: Photosynthetic CO_2_ assimilation
Ci: Internal CO_2_ concentration
CT: Computed tomography
DAP: Days after planting
FOV: Field of view
GBq: Gigabecquerel
KBq: Kilobecquerel
LOR: Line of response
MRI: Magnetic resonance imaging
MBq: Megabecquerel
PET: Positron emission tomography
ROI: Region of interest
TOA: Time of arrival
