# Supplementary figures and images for: In vivo quantitative imaging of photoassimilate transport dynamics and allocation in large plants using a commercial positron emission tomography (PET) scanner
Source: BMC Plant Biol. 2015 Nov 9;15:273. doi: 10.1186/s12870-015-0658-3 (PMC4640171; doi:10.1186/s12870-015-0658-3)

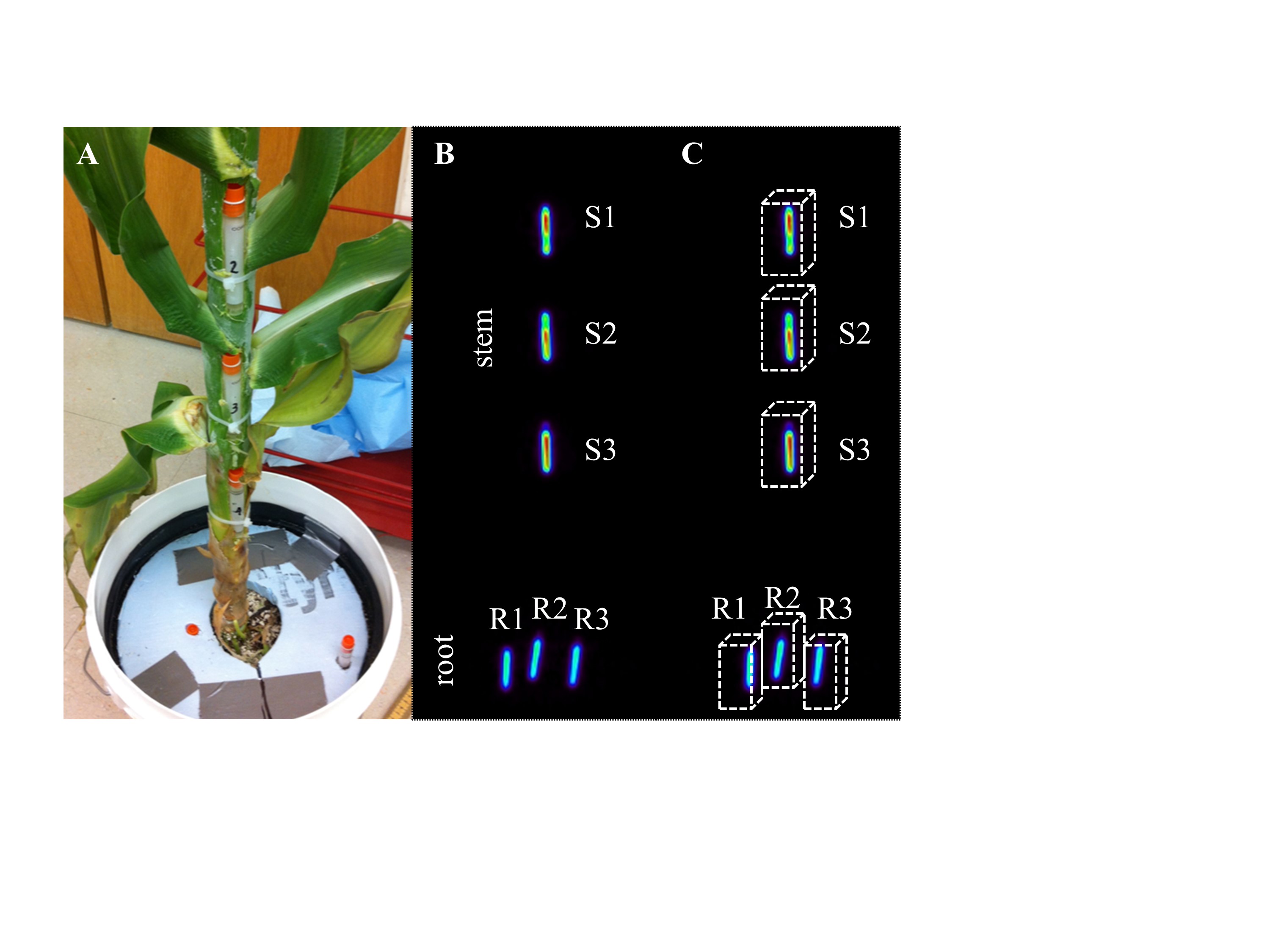

Supplement: Additional file 1: — 18 F phantoms used for analysis of attenuation and scatter factors. (A) The arrangement of 18F phantoms on the stem of the sorghum plant and inside the soil. (B) Image of the phantoms reconstructed from the PET emission scan. (C) ROIs outlined on the reconstructed image for quantification of radioactivity in each phantom vial. (JPG 377 kb) [file 12870_2015_658_MOESM1_ESM.jpg]

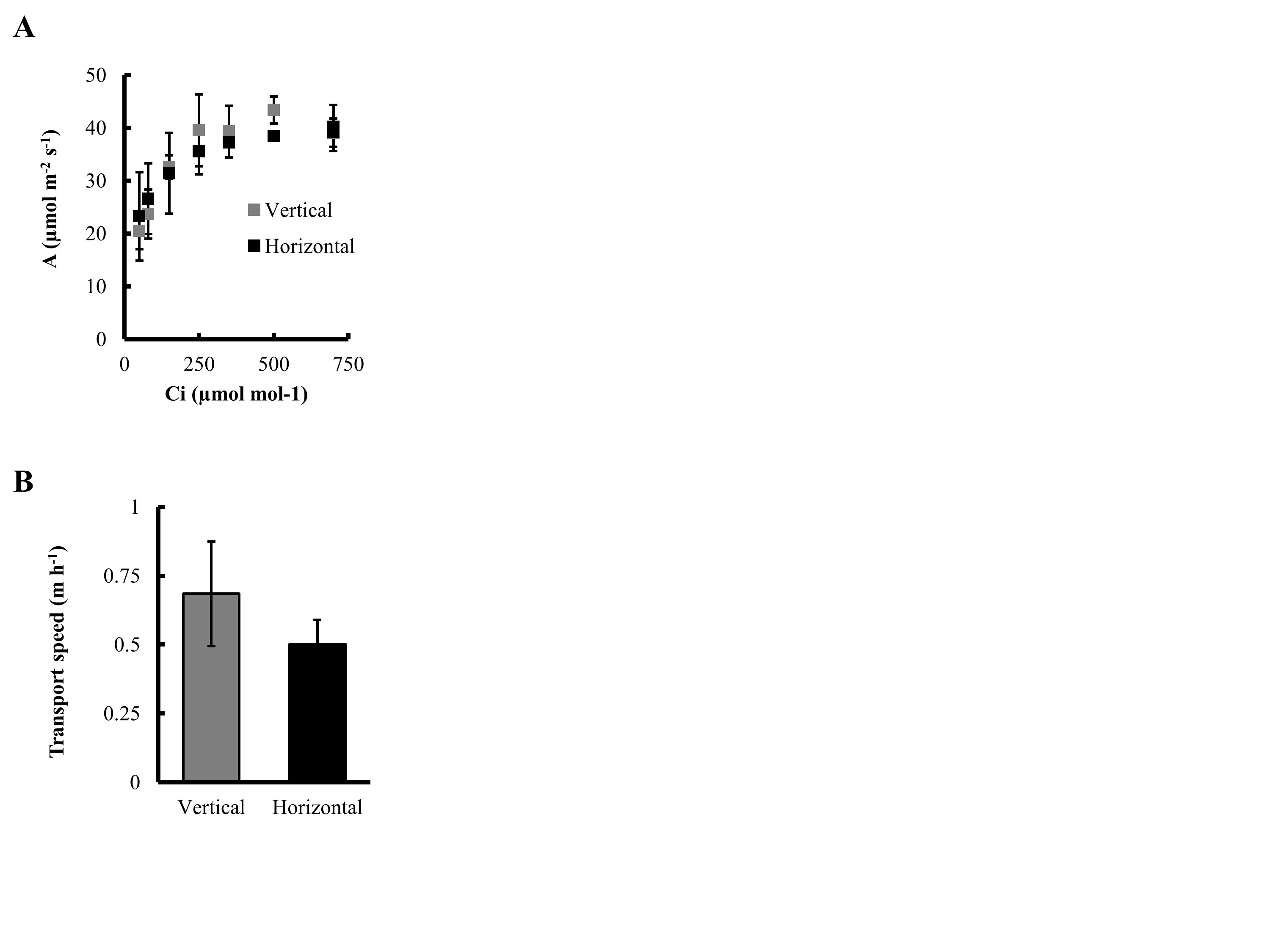

Supplement: Additional file 3: — Horizontal positioning has limited effects on physiological parameters of sorghum. (A) A/Ci curve of sorghum plant kept upright (vertical) or after laying it down (horizontal) for 2 h. The photosynthetic CO2 assimilation [A] was measured using a LI-6400XT portable photosynthesis system (Li-Cor, Lincoln, NE). All the measurements were performed at 2000 μmol m−2 s−1 light intensity in a leaf cuvette maintained at 30 °C. Internal CO2 concentration [Ci] of the leaf cuvette was programmed to adjust from 0–700 μmol mol−1 of CO2, at a flow rate of ~500 ml min−1, and relative humidity between 50-60 %. Data was recorded on the first fully expanded leaf (youngest leaf with collar fully open). (B) Transport speeds of vertically and horizontally positioned 40 DAP sorghum plants. For sorghum plants in the horizontal position, transport speeds were determined as described in the methods. For sorghum plants in the upright position, 11CO2 was administered as described previously [37]. Briefly, two γ-radiation detectors with collimated lead shielding were positioned on the stem of the sorghum below the point of attachment of the load leaf and data was acquired for 2 h. The transport speed was determined from the time-activity curve generated for each detector as previously described, dividing the distance between the two detectors by the time of transit of [11C]-photoassimilate between the two detectors [26, 39]. (JPG 188 kb) [file 12870_2015_658_MOESM3_ESM.jpg]
